# Multiplex CRISPR/Cas9 Editing of Rice Prolamin and GluA Glutelin Genes Reveals Subfamily-Specific Effects on Seed Protein Composition

**DOI:** 10.3390/plants14152355

**Published:** 2025-07-31

**Authors:** María H. Guzmán-López, Susana Sánchez-León, Miriam Marín-Sanz, Francisco Barro

**Affiliations:** Laboratory of Functional Genomics, Department of Plant Breeding, Institute for Sustainable Agriculture (IAS-CSIC), 14004 Córdoba, Spain; ssanchez@ias.csic.es (S.S.-L.); mmarin@ias.csic.es (M.M.-S.); fbarro@ias.csic.es (F.B.)

**Keywords:** prolamin, glutelin, rice, CRISPR, seed storage proteins

## Abstract

Rice seed storage proteins (SSPs) play a critical role in determining the nutritional quality, cooking properties, and digestibility of rice. To enhance seed quality, CRISPR/Cas9 genome editing was applied to modify SSP composition by targeting genes encoding 13 kDa prolamins and type A glutelins. Three CRISPR/Cas9 constructs were designed: one specific to the 13 kDa prolamin subfamily and two targeting conserved GluA glutelin regions. Edited T_0_ and T_1_ lines were generated and analyzed using InDel analysis, SDS-PAGE, Bradford assay, and RP-HPLC. Insertions were more frequent than deletions, accounting for 56% and 74% of mutations in prolamin and glutelin genes, respectively. Editing efficiency varied between sgRNAs. All lines with altered protein profiles contained InDels in target genes. SDS-PAGE confirmed the absence or reduction in bands corresponding to 13 kDa prolamins or GluA subunits, showing consistent profiles among lines carrying the same construct. Quantification revealed significant shifts in SSP composition, including increased albumin and globulin content. Prolamin-deficient lines showed reduced prolamins, while GluA-deficient lines exhibited increased prolamins. Total protein content was significantly elevated in all edited lines, suggesting enrichment in lysine-rich fractions. These findings demonstrate that CRISPR/Cas9-mediated editing of SSP genes can effectively reconfigure the rice protein profile and enhance its nutritional value.

## 1. Introduction

Rice (*Oryza sativa*) is the third most widely cultivated cereal in the world and serves as the staple food for over half of the world’s population [1,2,3]. After starch, seed storage proteins (SSPs) are the most abundant component in rice seeds, accounting for approximately 7–10% of the seed weight [4]. Rice SSPs are classified by solubility into four groups: albumins, globulins, prolamins, and glutelins, with their relative proportions varying among cultivars [3,5]. Among these, glutelins are the major fraction, while albumins, globulins, and prolamins are considered minor components.

Although prolamins are the least abundant SSP fraction, the rice genome (*O. sativa* cv. Nipponbare) contains 34 prolamin genes, classified into 10, 13, and 16 kDa groups, with 4, 28, and 2 genes copies, respectively [6]. Within these, the 13 kDa prolamins—subdivided into four subfamilies—are the most predominant but have a low lysine content, making them less nutritionally valuable compared to other prolamins rich in sulfur-containing amino acids [1,3,7].

Glutelins, by contrast, are considered high-quality proteins due to their superior nutritional profile relative to other cereal SSPs such as those in wheat [8]. Rice glutelins are synthesized as 57 kDa precursors and later processed into mature 37 kDa acidic (α) and 20 kDa basic glutelin (β) subunits. The 15 glutenin genes identified in rice are grouped into GluA, GluB, GluC, and GluD families [9]. Among these, GluA glutelins—despite being the most highly expressed—offer limited nutritional benefits compared to the GluB glutelins, which have higher lysine content and oligomerization capacity that may enhance processing qualities [10,11].

Rice SSPs lack the immunogenic epitopes present in wheat gluten, making them suitable for gluten-free diets [12]. However, unlike wheat gluten, rice proteins do not form a cohesive network, often requiring the addition of other ingredients that compromise the nutritional quality of gluten-free products [13]. Additionally, the SSP content significantly affects the cooking behavior, digestibility, and nutritional value of rice-based foods [14,15]. Therefore, enhancing the quality and balance of these proteins could improve both the functionality and nutritional profile of rice and its gluten-free derived products.

Previous studies have employed genetic engineering, particularly RNA of interference (RNAi), to reduce the accumulation of specific SSPs—namely prolamins and glutelins—and evaluate their impact on seed protein composition [8,16,17,18,19,20]. These studies revealed compensatory mechanisms between protein fractions; for instance, a reduction in glutelins often triggered increases in prolamins, globulins, and other SSPs [8,20,21]. Likewise, silencing 13 kDa prolamins has been associated with elevated levels of glutelins and globulins [8].

More recently, CRISPR/Cas9 has emerged as a powerful tool for precise genome editing in crops, enabling targeted modification without transgene integration, to introduce new traits in crops [22]. In rice, CRISPR/Cas9 has been used to edit individual or multiple glutelin genes [21,23,24,25], and more recently, to target Pro13a and Pro13b prolamin families independently [26]. Pham et al. [26] reported reciprocal compensation between these two prolamin subfamilies when edited separately. For glutelins, several studies have targeted members of different subfamilies [23,24,25], while only one study focused on GluA genes by designing sgRNAs against the fourth exon of GluA-1 and GluA-2 [21]. However, to our knowledge, no studies have simultaneously edited both the Pro13a and Pro13b genes or specifically targeted the acidic subunit of GluA glutelins, located within the first two exons and part of the third exon.

This study describes the development of CRISPR/Cas9-edited rice lines in which either all 13 kDa prolamins or GluA glutelins—both associated with lower nutritional quality—were disrupted. The resulting lines exhibit altered SSP profiles with potentially improved functional and nutritional properties. Additionally, our study contributes to understanding compensatory response among SSP fractions and provides a basis for future improvement in rice composition through targeted editing.

## 2. Materials and Methods

### 2.1. Sanger Sequencing of Prolamin and Glutelin Genes in the EYI105 Cultivar

Genomic sequences of prolamin and glutelin genes were obtained from the rice cultivar EYI105 using primers designed based on annotated sequences from the Nipponbare genome [6,9] (Supplemental Table S1). These primers successfully amplified 27 out of 28 prolamin genes and all three GluA glutelin genes described in Nipponbare. The resulting PCR amplicons were cloned into the pGEMT Easy vector (Promega, Madison, WI, USA) and transformed into *Escherichia coli* DH5α. Sanger sequencing was performed by STABVIDA (Caparica, Portugal), and sequence assemblies were generated using Geneious Prime^®^ 2023.1.1 software (https://www.geneious.com, accessed on 14 August 2020).

### 2.2. Designing and Assembly of CRISPR/Cas9 Constructs

Reference contigs for 13 kDa prolamins and GluA glutelins in EYI105 were assembled from the Sanger sequencing data using the default settings in Geneious. To assess whether these contigs captured the genetic diversity present in EYI105, they were compared with the annotated gene sets of Nipponbare. Phylogenetic analysis was performed using MEGA v10.2.6 and maximum-likelihood methods [27].

Based on the assembled contigs, six single-guide RNAs (sgRNAs) were designed to target conserved regions of prolamin and GluA genes (Table 1). sgRNA selection was guided by predicted activity scores calculated using the Doench et al. [28] model implemented in Geneious, which evaluates sequence features near the target site to estimate editing efficiency. Additionally, sgRNAs were chosen for their ability to target multiple genes within the GluA glutelin family and especially within the Pro13a and Pro13b prolamin subfamilies, maximizing the editing effect on these multigene families.

To ensure compatibility with the rice U6 promoter, some sgRNAs were modified by adding a 5′ guanine nucleotide. Potential off-targets were evaluated by searching sgRNA sequences against the coding regions (CDSs) of release 7 of the Rice Genome Annotation Project (RGAP), considering only hits with PAM motifs and up to two mismatches outside the PAM site [29,30].

**Figure 1:**
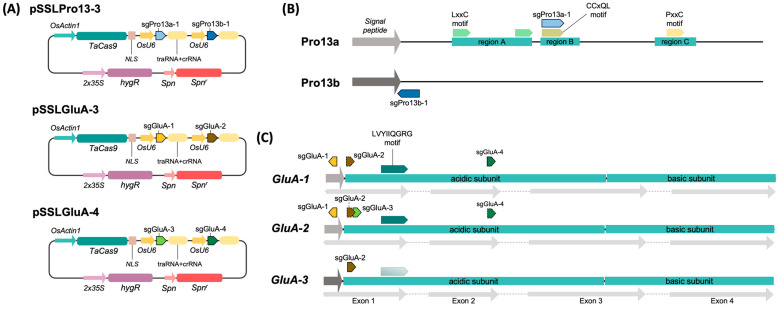
Expression vectors and CRISPR/Cas9 target sites in Nipponbare prolamin and GluA gene models. (**A**) Schematic diagram of the expression vectors used for plant transformation. (**B**) Schematic diagram of typical Pro13a and Pro13b Nipponbare prolamins highlighting the conserved prolamin regions named A, B, and C and their characteristic protein motifs LxxC, CCxQL, and PxxC [2]. Target sequences for the sgRNAs are represented by colored arrows. (**C**) Schematic diagram of GluA genes described in Nipponbare highlighting their CDS and their acidic and basic subunits. Target sequences for the sgRNAs are represented by colored arrows. The LVYIIQGRG motif, essential for the correct assembly of proglutelins, is also depicted [32]. Lighter arrows or dashed lines indicate that the sequence is present with 1 or 2 mistmatches.

The sgRNAs were assembled into three CRISPR/Cas9 expression vectors: pSSLPro13-3 (targeting Pro13a and Pro13b prolamins), and pSSLGluA-3 and pSSLGluA-4 (targeting the acidic subunit of GluA glutelins). Each construct included a codon-optimized Cas9 under the control of the *Oryza sativa* Actin1 promoter (OsAct1) (Figure 1A), and two sgRNAs driven by independent *O. sativa* U6 promoters (OsU6). Vector assembly was performed using the modular toolkit described by Čermák et al. [31], using the pMOD_A1111, pMOD_B2520, pMOD_C2520 and pTRANS_110 from the Voytas Lab Plant Genome Engineering Toolkit (http://crispr-multiplex.cbs.umn.edu, accessed on 10 August 2020).

### 2.3. Rice Transformation and Plant Growth Conditions

The rice transformation was carried out following the protocol described by Baysal et al. [33], with minor modifications. Mature zygotic embryos from 7-day-old seeds of *O. sativa* cv. EYI105 were isolated and pre-incubated for 4 h on Murashige and Skoog (MS) osmotic medium prior to bombardment. Embryos were subjected to two rounds of particle bombardment at a 4 h interval, as previously described by Pistón et al. [34]. A single CRISPR/Cas9 construct was used per transformation event; no co-transformation was carried out.

After overnight incubation in darkness, embryos were transferred to MS medium with 50 mg/L hygromycin and 2.5 mg/L 2,4-dichlorophenoxyacetic acid, and incubated for 2–3 weeks in the dark [35]. Developing embryogenic calluses were then moved to the regeneration medium, and upon shoot emergence, transferred to rooting medium. Regenerated plantlets were acclimated and grown to maturity in soil under greenhouse conditions (28/25 °C day/night, 12 h photoperiod) with supplementary light when necessary. Plants were irrigated with tap water containing 100 μM Fe(III)-EDDHA (Sequestrene 138 Fe G-100; Syngenta Agro, Madrid, Spain).

### 2.4. DNA Extraction and PCR Analysis

Young leaf tissue was harvested from T0 and T1 plants, ground in liquid nitrogen, and stored at −80 °C until DNA extraction. Genomic DNA was isolated using the CTAB method described by Murray and Thompson [36].

To detect the presence of the *Cas9* gene, PCR amplification was performed using the primers listed in Supplemental Table S1. Each 25 µL reaction contained 100 ng of genomic DNA, 400 nM of each primer, 320 µM dNTPs, and 0.65 units of Taq DNA polymerase (Biotools, Madrid, Spain). The thermal cycling conditions were as follows: initial denaturation at 95 °C for 3 min; 35 cycles of 95 °C for 15 s, 58 °C for 30 s, and 72 °C for 1 min; followed by a final extension at 72 °C for 7 min. PCR products were visualized by electrophoresis on a 1% agarose gel.

### 2.5. InDels Characterization

To detect insertions and deletions (InDels) in edited plants, PCR amplification of the target gene regions was performed using the primers listed in Supplemental Table S1. The resulting amplicons were subjected to Sanger sequencing. De novo assembly of the sequencing reads was carried out using Geneious Prime^®^ 2023.1 software. Aligned sequences were compared to the reference contigs for 13 kDa prolamins and *GluA* glutelins previously generated from the EYI105 genotype (see Section 2.2), to identify on-target InDels in each putative edited line.

### 2.6. Polyacrylamide Gel Electrophoresis Analysis (PAGE)

The SSP profile was characterized in each line using the half-seed technique, which preserves the embryo for plant propagation while using the endosperm for protein extraction. The endosperm half-seed was ground into a fine powder and a modified buffer from Kawakatsu et al. [9] containing 50 mM Tris-HCl (pH 6.8), 4% SDS, 2 M urea, 20% glycerol, 2% dithiothreitol (DTT), and 0.002% bromophenol blue added at a ratio of 20:1 (μL mg^−1^ flour). Samples were vortexed and incubated at room temperature with continuous shaking for 45 min, then centrifuged at 13,000× *g* for 10 min to remove debris. The supernatants were loaded and separated in SDS-PAGE using either 12% Criterion™ TGX™ precast midi gels (BIO-RAD, Hercules, CA, USA) or self-made SDS-PAGE gels (stacking: T = 12%, C = 2.67%; loading: T = 4%, C = 2.67%) and run for 5 h at 10 °C and 20 mA per gel. Protein bands were visualized by staining with a solution containing 0.05% (*w*/*v*) Coomassie Blue R-250, 5% (*v*/*v*) ethanol, and 4% (*w*/*v*) trichloroacetic acid.

### 2.7. Determination of Albumins and Globulins

Albumin and globulin fractions were sequentially extracted from 50 mg of rice flour. Albumins were extracted stepwise three times with 250 μL of ultrapure water, vortexing for 2 min and incubation at room temperature (RT) for 15 min with shaking. After centrifugation at 13,000× *g* for 10 min, supernatants from all three steps were pooled. Globulins were extracted from the pellet using the same procedure but replacing water with 250 μL 0.5 M NaCl and also pooling supernatants. All extractions were carried out in quadruplicate.

The protein concentrations were determined using the Bradford assay (Reagent (B-6916, Sigma-Aldrich, San Luis, MO, USA), with bovine serum albumin (BSA; 0332, VWR, West Chester, PA, USA) as the standard. Absorbance was measured at 595 nm using a microplate reader.

### 2.8. Reversed-Phase High-Performance Liquid Chromatography (RP-HPLC) of Prolamins and Glutelins

Prolamin and glutelin fractions were sequentially extracted from the pellet remaining after globulin extraction. Prolamins were extracted stepwise twice with 400 μL of 60% 1-propanol, vortexing for 2 min and incubation at RT for 30 min with shaking. After each extraction, samples were centrifuged at 15,000× *g* for 10 min, and supernatants pooled. Glutelins were then extracted from the insoluble pellet stepwise three times with 335 μL of extraction buffer containing 50% (*v*/*v*) 1-propanol, 2 M urea, 0.05 M Tris–HCl (pH 7.5), and 2% (*w*/*v*) DTT. Each step involved vortexing for 2 min and incubation at 60 °C for 15 min with shaking. After centrifugation at 6000× *g* for 20 min, supernatants were pooled. All extractions were carried out in quadruplicate.

Before RP-HPLC analysis, samples were filtered using 0.45 µm Costar^®^ Spin-X^®^ centrifuge tube filters (Corning Inc., Corning, NY, USA). Prolamin (20 μL) and glutelin (22 μL) extracts were analyzed using a 300SB-C8 reverse-phase analytical column (4.6 × 250 mm, 5 μm particle size, 300 Å pore size; Agilent Technologies, Santa Clara, CA, USA) on a 1200 Series Quaternary LC System (Agilent) as described in Ozuna and Barro [37]. Chromatograms were processed using OpenLab CDS 2.8 software with minor manual adjustments. Quantification was performed using BSA as the standard.

### 2.9. Statistical Analysis

All statistical analyses were performed using the R software [38]. Comparisons between two groups were carried out using either Student’s *t*-test or Welch’s *t*-test for normally distributed data, and the Mann–Whitney Wilcoxon test for non-parametric data.

## 3. Results

### 3.1. Multiplex Genome Editing of Rice Prolamins and Glutelins by CRISPR/Cas9

To design effective CRISPR/Cas9 constructs, the 13 kDa prolamin and *GluA* glutelin gene families were sequenced in the rice cultivar EYI105, providing 60 and 30 unique sequences, respectively (Supplemental Table S2). These sequences, along with annotated genes from the Nipponbare reference genome [6,9] were assembled into six 13 kDa prolamin and four GluA glutelin contigs (Supplemental Table S3), which were used as references for sgRNA design and InDels detection.

**Figure 2:**
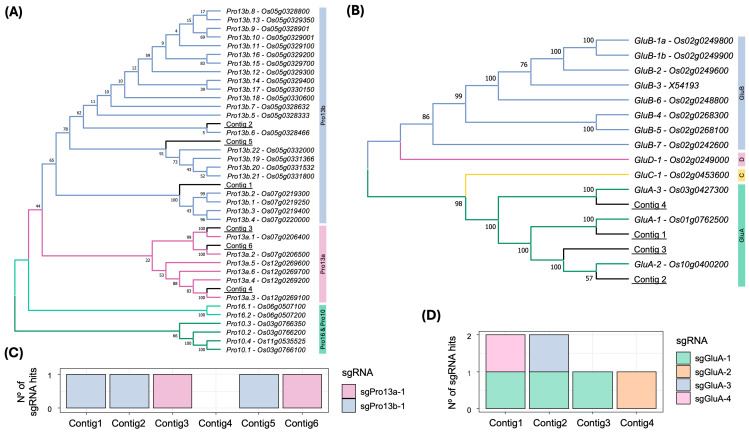
Phylogenetic comparison between EYI105 contigs and Nipponbare genes, and distribution of sgRNAs in 13 kDa prolamin and GluA sequences. (**A**) Maximum-likelihood tree depicting the 13 kDa prolamin contigs identified in the EYI105 rice cultivar in this study, alongside prolamin genes described in Nipponbare by Saito et al. [6]. Each prolamin gene name is accompanied by its Rice Annotation Project Database (RAP-DB) identifier [30,40]. (**B**) Maximum-likelihood tree depicting the GluA contigs identified in EYI105 in the present study, alongside glutelin genes described in Nipponbare [9]. Each glutelin gene name is accompanied by its RAP-DB identifier, except for the GluB-3 pseudogene, which is accompanied by its GenBank identifier. (**C**) Number of sgRNAs (without mismatches) targeting the 13 kDa prolamin contigs identified in EYI105 rice cultivar. (**D**) Number of sgRNAs (without mismatches) targeting the glutelin A contigs identified in EYI105 rice cultivar.

Phylogenetic analysis confirmed the identity of these contigs, which clustered with their corresponding Nipponbare genes (Figure 2A,B). Prolamin contigs 3, 4, and 6 aligned with Pro13a genes from Nipponbare, whereas contigs 1, 2, and 5 grouped with Pro13b genes. For glutelin, contigs 1 and 4 were linked to *GluA-1* and *GluA-3* while *GluA-2* was represented by contigs 1 and 2.

Based on these alignments, six sgRNAs were designed to target conserved coding regions of the 13 kDa prolamins and *GluA* glutelins (Figure 1B–C, Supplemental Figures S1 and S2). Specifically, sgPro13a-1 targeted the conserved CCxQL motif [39] in Pro13a, while sgPro13b-1 targeted the signal peptide region of Pro13b. For GluA genes, sgRNAs targeted the signal peptide (sgGluA-1), exon 1 (sgGluA-2 and sgGluA-3), and a site downstream of the LVYIIQGRG motif in exon 2 (sgGluA-4), essential for proglutelin assembly and ER export [32].

All Pro13a contigs were putatively targeted by the sgRNA sgPro13a-1, and all Pro13b contigs by sgPro13b-1 (Figure 2C), except for contig 4 (*Pro13a.3*), which lacked both sgRNA sequences. Regarding glutelins, contig 1, 2, and 4 were targeted by sgGluA-4, sgGluA-3, and sgGluA-2, respectively (Figure 2D). Notably, sgGluA-1 matched contigs 1, 2, and 3, while sgGluA-2 also matched contigs 1, 2, and 3 with two mismatches, and sgGluA-4 was present in contigs 2 and 3 with a single mismatch in the seed sequence (Figures S1–S3). No off-target sites were detected in the CDS of rice genes.

The sgRNAs were combined into three CRISPR/Cas9 expression vectors (Table 1): pSSLPro13-3 (for Pro13a/Pro13b), pSSLGluA-3, and pSSLGluA-4 (for GluA glutelins). These constructs were independently introduced into EYI105 scutella, resulting in 164, 123, and 15 regenerated T0 lines, respectively. A total of 210 T0 plants were confirmed as Cas9-positive by PCR (Table 2). T1 seeds were obtained from 55% of pSSLPro13-3, 72% of pSSLGluA-3, and 100% pSSLGluA-4 of Cas9-positive lines.

### 3.2. Genome Editing of Prolamin and Glutelin Genes Provides Distinctive Protein Profiles

To evaluate the phenotypic impact of CRISPR/Cas9 editing, total seed storage proteins (SSPs) were extracted from T0 seeds and analyzed by SDS-PAGE. In total, 610 half-seeds were screened from 36, 27, and 5 T0 lines derived from pSSLPro13-3, pSSLGluA-3, and pSSLGluA-4, respectively. Protein profile alterations were observed in 27 lines from pSSLPro13-3, 21 from pSSLGluA-3, and all 5 from pSSLGluA-4 (Table 2). Segregating patterns were detected in 18% of prolamin-edited and 20% of glutelin-edited lines from pSSLGluA-3. These lines exhibited both the wild type and a single, consistent altered protein pattern among the grains analyzed for each plasmid. In contrast, all lines transformed with pSSLGluA-4 showed altered and stable non-segregating profiles.

**Figure 3:**
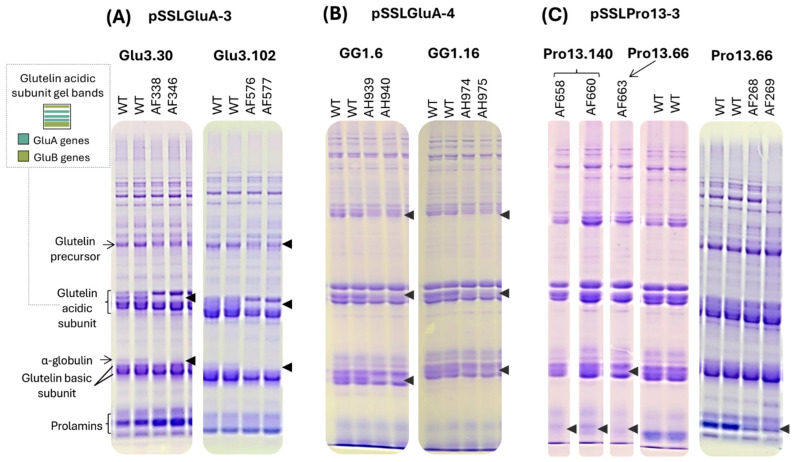
Examples of SDS-PAGE total SSP profile of T1 half-seeds derived from T0 lines transformed with (**A**) pSSLGluA-3, (**B**) pSSLGluA-4, (**C**) pSSLPro13-3. The arrows indicate the absence of bands in edited lines. A schematic representation of the gel bands of glutelin acidic subunits, as described in Katsube-Tanaka et al. [11], is depicted in a black box with dashed lines. SSP: seed storage protein. WT: wild type.

All edited lines transformed with the same CRISPR/Cas9 construct exhibited consistent protein profiles (Figure 3). In lines edited with pSSLPro13-3, SDS-PAGE showed the absence of the 13 kDa prolamin bands, confirming effective disruption of both *Pro13a* and *Pro13b* genes (Figure 3C). Notably, one T1 descendant (AF663) of the Pro13.66 line, also lacked bands corresponding to glutelin subunits.

Lines edited with pSSLGluA-3 and pSSLGluA-4 lacked bands corresponding to GluA-derived acidic subunits (Figure 3A,B) [11], as well as deficiencies in specific bands corresponding to glutelin basic subunits, along with reduced intensity in the protein bands corresponding to the glutelin precursor. The most pronounced alterations were observed in pSSLGluA-3-derived lines, which also lacked the α-globulin band. In contrast, pSSLGluA-4 lines retained faint bands corresponding to glutelin precursors. Due to the high consistency in protein profiles among edited lines derived from the same construct, additional Cas9-positive lines were not further analyzed by SDS-PAGE.

To confirm inheritance and profile stability, 42 Prolamin and 18 Glutelin edited T1 lines from pSSLPro13-3 and pSSLGluA-3 plasmids and were grown and self-pollinated to produce the T2 seeds. Cas9-negative segregants were identified by PCR in 35% (Prolamin) and 27% (Glutelin) T1 lines (Table 3). A subset of 15 Prolamin-edited and 11 Glutelin-A-edited T2 lines were analyzed by SDS-PAGE, all of which showed protein profiles identical to their T1 parent lines (Figure S4), suggesting stable homozygous mutations.

### 3.3. Alterations in the Protein Profile Are Consequences of InDels in Prolamin and Glutelin Genes

**Figure 4:**
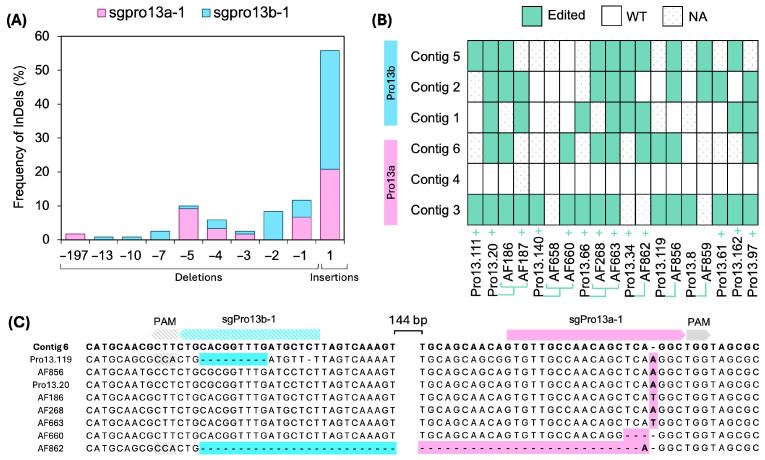
Analysis of InDel patterns and heritability in CRISPR/Cas9-edited 13 kDa prolamin genes. (**A**) Frequency of InDels in Sanger clones, expressed as a percentage, and calculated by dividing the total events within each mutation length interval by the overall number of mutations identified in 13 kDa prolamins of pSSLPro13-3 lines. (**B**) The heritability of mutations in 13 kDa prolamins across generations of edited lines. WT: wild type. NA: not applicable, no clones retrieved. +: Cas9 positive line. T1 lines are linked by green lines to their T0 parental line. InDels: insertions and deletions. (**C**) Illustrative examples of insertions in sgPro13a-1 and sgPro13b-1 in contig 6 of EYI105 prolamins.

To validate the molecular basis of the observed SSP profile alterations, selected edited T_0_ and T_1_ lines were analyzed by Sanger sequencing to identify InDels at the target loci. A total of 19, 17, and 5 lines derived from pSSLPro13-3, pSSLGluA-3, and pSSLGluA-4, respectively, were analyzed. Across all samples, 357 unique sequence clones were generated and aligned to the corresponding EYI105 prolamin or GluA contigs (Supplemental Table S2). In all lines showing altered SDS-PAGE profiles, on-target InDels were detected, establishing a clear correlation between genotype and protein phenotype. The only exception was line AF658, for which all clones were mapped to contigs 2 and 4 of the Pro13b family and lacked detectable mutations (Figure 4B).

**Figure 5:**
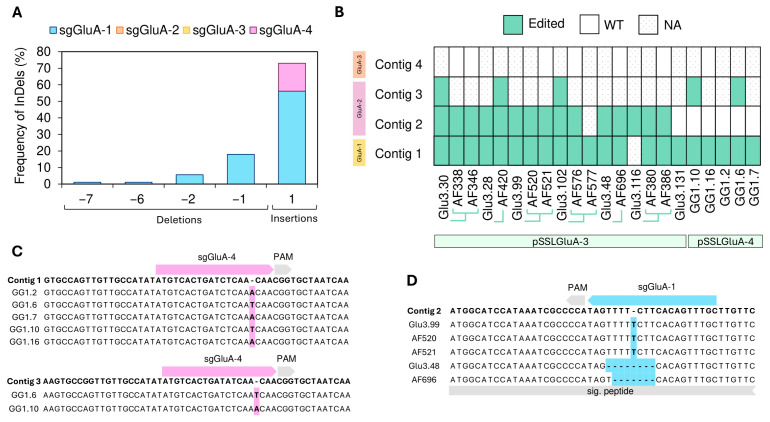
Analysis of InDel patterns and heritability in CRISPR/Cas9-edited type A glutelin genes. (**A**) Frequency of InDels in Sanger clones, expressed as a percentage, and calculated by dividing the total events within each mutation length interval by the overall number of mutations identified in GluA glutelins of pSSLGluA-3 and pSSLGluA-4 lines. (**B**) The heritability of mutations in GluA glutelins across generations of edited lines. WT: wild type. NA: not applicable, no clones retrieved. T1 lines are linked by green lines to their T0 parental line. InDels: insertions and deletions. (**C**) Illustrative examples of insertions in sgGluA-4 in contigs 1 and 3 of EYI105 glutelins. (**D**) Illustrative examples of insertions in sgGluA-1 in contig 2 of EYI105 glutelins.

Insertions were more frequent than deletions, accounting for 56% of mutations in prolamins and 74% of glutelins (Figure 4A and Figure 5A). In all cases, insertions were 1-base pair (bp) in length, providing typical Cas9 frameshifts. For instance, sgGluA-4 (in pSSLGluA-4) exclusively generated +1 bp insertions (Figure 5C). Deletions produced by sgGluA-1 (in pSSLGluA-3) ranged from −1 to −7 bp, with the −1 bp being the most prevalent (Figure 5A,D). The only in-frame deletion (−6 bp) was observed in the AF696 T1 line in contig 2.

For prolamins, deletions sizes ranged from −1 to −197 bp (Figure 4A). The most frequent deletions were -5 bp for sgPro13a-1 and −2 bp for sgPro13b-1 (Figure S5). The largest deletion (−197 bp) overlapped the cleavage sites of both sgPro13a-1 and sgPro13b-1 on contig 6, detected in line AF862 (Figure 4C). Although contig 6 lacks a perfect match for sgPro13b-1, both the AF862 sequence and the Pro13.119 sequence retained the PAM site seed sequence (Figure 4C), which likely enable editing. Overall, 96.6% of prolamin mutations induced frameshifts (Figure 4A).

The efficiency of each sgRNA showed a wide range of variation. In prolamin-edited lines, sgPro13b-1 and sgPro13a-1 were responsible for 57% and 43% of the observed edits, respectively (Figure S6A). In glutelin-edited lines, only one sgRNA per construct was active: sgGluA-1 (pSSLGluA-3) and sgGluA-4 (pSSLGluA-4) (Figure 5A and Figure S6B). Between these two sgRNAs, sgGluA-1 demonstrated higher efficiency, with 84% of sequences exhibiting edits at its site, compared to 48% for sgGluA-4.

The inheritance and distribution of mutations across prolamin and glutelin contigs were examined (Figure 4B and Figure 5B). For glutelins, multiple lines derived from the pSSLGluA-3 and pSSLGluA-4 vectors, were frequently edited, particularly contigs 1, 2, and 3 (Figure 5B). In prolamins, Pro13b contigs were edited in various lines (Pro13.20, AF663, and Pro13.34), while all editable Pro13a contigs were edited in several lines (Pro13.20 and its AF186 descendant, AF660, AF268 and its related AF663 T1 line, Pro13.119 and its AF856 descendant, and Pro13.97) (Figure 4B). Notably, AF663 and Pro13.20 showed edits across all targets (Figure 4B).

Although Sanger sequencing of individual clones does not confirm zygosity, several lines exhibited uniform edits across all retrieved sequences for specific contigs, suggesting homozygosity (Supplemental Tables S4 and S5). The incomplete recovery of all possible edited alleles may have led to an underestimation of editing frequency.

### 3.4. Quantification of SSP Fractions in Edited Plants

To assess the impact of gene editing on protein composition, SSP fractions were quantified in lines showing uniform altered profiles across all half-seeds tested by SDS-PAGE. These lines were derived from pSSLPro13-3 and pSSLGluA-3 (T1), and from pSSLGluA-4 (T0). Albumins and globulins were quantified using the Bradford assay, while prolamins and glutelins were analyzed by RP-HPLC. As controls, nine lines were included: four wild type (WT) EYI105 plants and five Cas9-negative segregants that had undergone transformation and self-pollination.

**Figure 6:**
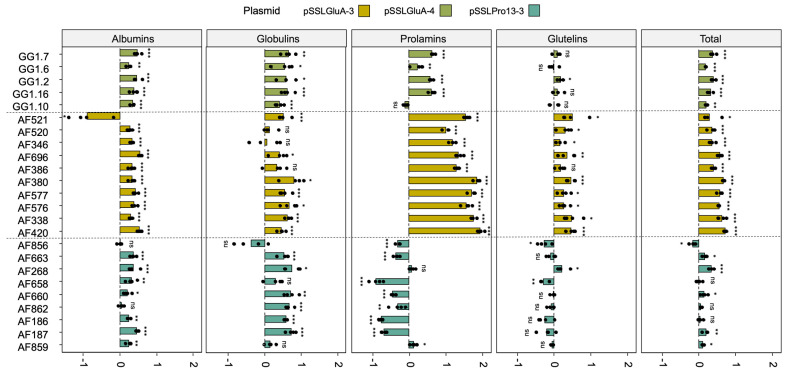
Albumin, globulin, prolamin, and glutelin protein fractions fold-change (log2(FC)) relative to the EYI105 control line of edited T1 lines derived from the pSSLGluA-3, pSSLGluA-4, and pSSLPro13-3 constructs. Protein fractions were extracted sequentially and quantified using the Bradford assay (for albumins and globulins) and RP-HPLC (for prolamins and glutelins). Total protein content was calculated as the sum of all fractions. The statistical analysis for each protein fraction was performed by the non-parametric Mann–Whitney–Wilcoxon test or by the *t*-test/Welch’s *t*-test, when normally distributed. *p* < 0.05, *; *p* < 0.01, **; *p* < 0.001, ***; ns, non-significant.

Edited lines exhibited significant changes in protein composition compared to WT controls (Figure 6). Across constructs, most lines showed increased albumin and globulin contents. Notably, this increase was accompanied by a significant reduction in the prolamin fraction in lines derived from the pSSLPro13-3 construct (Figure 6). An exception was line AF268, which showed no significant change in total prolamin content but did present a distinct RP-HPLC peak pattern compared to WT (Figure S7).

Glutelin-targeted lines (pSSLGluA-3 and pSSLGluA-4) showed a significant increase in prolamins, particularly in those edited with pSSLGluA-3 (Figure 6 and Figure S7). However, glutelin content varied between constructs. Most pSSLGluA-3 lines displayed significantly elevated glutelin levels, whereas pSSLGluA-4 lines generally showed no significant difference compared to the WT.

Among lines targeted with pSSLPro13-3 construct, three lines (AF856, AF658, and AF268) showed altered glutelin levels; AF856 and AF658 showed a reduction, while AF268 exhibited an increase. Overall, most edited lines presented a significant increase in total protein content, especially those edited in *GluA* glutelin genes.

**Figure 7:**
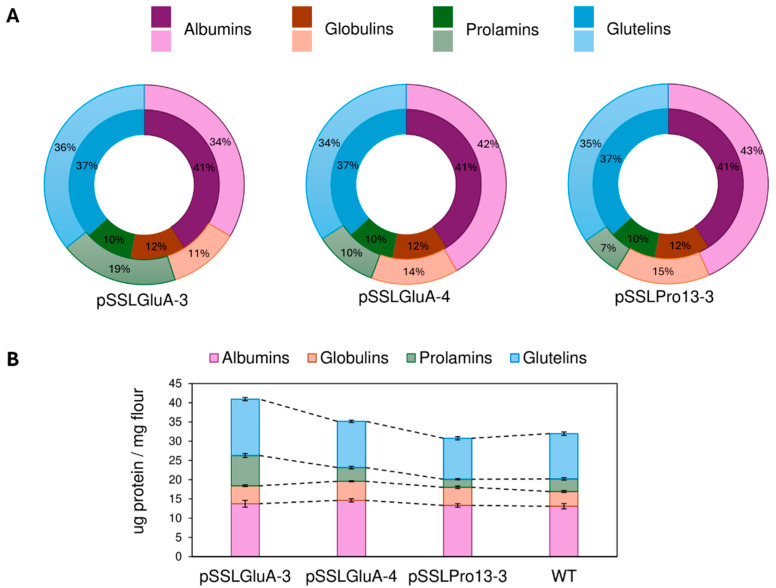
Comparison of seed storage protein fractions in wild type and CRISPR-edited rice lines derived from pSSLGluA-3, pSSLGluA-4, and pSSLPro13-3 constructs. (**A**) Median percentages of albumin, globulin, prolamin, and glutelin fractions in edited lines derived from pSSLGluA-3, pSSLGluA-4, and pSSLPro13-3 constructs, relative to their respective total protein content. The inner circle denotes the median percentage of protein fractions in EYI105 control lines. (**B**) Average content of each protein fraction in the WT and in the edited lines derived from pSSLGluA-3, pSSLGluA-4, and pSSLPro13-3 constructs. WT: wild type.

In the case of pSSLGluA-4-derived lines, protein fraction proportions were analyzed relative to total SSP content. Despite absolute increases in several fractions, the relative proportions of albumins and globulins increased by 2% and 1%, respectively, compared to the WT lines, while prolamins remained unchanged. In contrast, pSSLGluA-3 lines exhibited pronounced relative increases in prolamins (+9%) and albumins (+7%), while globulins and glutelins remained proportionally stable. Prolamin-deficient lines showed an inverse trend: reduced proportions of prolamins (−3%) and glutelins (−2%), balanced by increases in globulins and albumins (+3% and +2%, respectively) (Figure 7).

## 4. Discussion

Rice stands as a crucial staple crop worldwide, renowned for its high-quality protein. However, its SSPs, while hypoallergenic, lack the functional properties of gluten and require the addition of functional agents that may compromise the nutritional value of gluten-free products [41]. In this context, modifying the protein composition of rice seeds may improve both functionality and nutritional quality.

In this study, CRISPR/Cas9 was used to target two SSP groups associated with low lysine content: 13 kDa prolamins and GluA glutelins. Target sites for sgRNAs were designed in the conserved coding regions of these genes. In the case of the 13 kDa prolamin genes, two sgRNA were designed: sgPro13a-1, targeting the coding sequence of the CCxQL motif in the Pro13a fraction [39], and sgPro13b-1 targeting the signal peptide of Pro13b fraction. This approach differs from that reported by Kawakatsu et al. [8] wherein the Pro13a and Pro13b fraction were targeted with an RNAi silencing fragment within the signal peptide and the 3′ UTR region, respectively. Other authors also reported RNAi silencing fragments to the conserved region of the signal peptide and a 505 bp region of the *Pro13a.2* gene [16,18]. A recent study also targeted prolamin genes using CRISPR/Cas9 but focusing on a conserved sequence within the coding regions of the prolamin genes [26]. However, Pro13a and Pro13b genes were not edited simultaneously, and compensatory effects were observed between the two 13 kDa prolamin subgroups.

For GluA genes, sgRNAs were targeting the signal peptide, and the first or the second exon. Previous studies have attempted to edit glutelin genes by CRISPR/Cas9 technology [21,23,24,25], but just one study focused on GluA genes, targeting the fourth exon of the *GluA-1* and *GluA-2* genes [21].

Our multiplex approach enabled the simultaneous editing of both Pro13a and Pro13b subfamilies, as well as specific disruption of GluA acidic subunits.

Analysis of T0, T1, and T2 lines confirmed that editing efficiency varied among sgRNAs, with certain guides consistently generating +1 bp insertions or short deletions that disrupted coding sequences. The edited lines exhibited distinct protein profiles, including the complete absence of 13 kDa prolamins or GluA-related bands, depending on the construct used. Notably, lines derived from pSSLGluA-3 exhibited a complete lack of protein bands related to GluA subunits, whereas those derived from pSSLGluA-4, generally, did not show such a reduction. This difference likely reflects the location of the sgRNA in the target sequences: while sgGluA-1 (pSSLGluA-3) targeted the signal peptide, sgGluA-4 (pSSLGluA-4) targeted a site downstream of the LVYIIQGRG motif, preserving regions essential for proper proglutelin processing [32]. Similar modifications in the SSP profile were observed in other studies in which either prolamin or glutelin genes were knocked down or silenced [8,16,17,18,21]. These protein profiles remained stable across generations, and InDel analysis corroborated the presence of frameshift mutations at the target sites in all edited lines with altered phenotypes. The exception was the AF658 T1 line, in which none of the 11 unique clones retrieved exhibited any edits. Notably, all clones from this line mapped to prolamin contigs 2 and 4, which also remained unedited in both the parental T0 line and a related T1 line. However, it is noteworthy that other prolamin contigs in the T0 and T1 relatives showed InDels, suggesting insufficient sequencing coverage as the reason for this discrepancy. Additionally, we hypothesize that the accumulation of edits in specific lines, such as the AF663 line, which exhibited edits across all prolamin contigs, may lead to the silencing of a larger number of prolamin genes compared to other lines.

The differential activity observed among sgRNAs in this study is consistent with previous reports using CRISPR/Cas9 in rice. For instance, Chandra et al. [21] and Pham et al. (2024) [26] also reported that only a subset of the designed sgRNAs provided detectable edits, with efficiency influenced by factors such as chromatin accessibility, target sequence composition, and position within the gene. In our constructs, only one sgRNA per GluA-targeting vector produced InDels (sgGluA-1 in pSSLGluA-3 and sgGluA-4 in pSSLGluA-4), while both prolamin-targeting guides were active. Notably, the glutelin guides that failed to induce edits (sgGluA-2 and sgGluA-3) overlapped within the 5′ coding region, suggesting that local DNA accessibility may have constrained Cas9 activity at those sites, as previously proposed by Chung et al. [42]. As stated previously, the study conducted by Chandra et al. [21] focused on targeting the *GluA-1* and *GluA-2* genes, successfully identifying InDels only in the *GluA-1* gene. In this study, InDels were successfully introduced in contigs associated with both the *GluA-1* and *GluA-2* genes.

Regarding mutation types, the predominance of +1 bp insertions and short deletions observed in our lines aligns with the typical outcomes of non-homologous end joining (NHEJ) in rice [25,42]. These studies also reported a bias toward +1 insertions when targeting conserved coding regions, particularly near the 5′ end of genes. In our case, all insertions were +1 bp, and the most frequent deletions ranged from −1 to −7 bp in glutelins and −2 to −5 bp in prolamins, with a few longer deletions (e.g., −197 bp) likely resulting from simultaneous cleavage at adjacent target sites. Although deletions are generally more common than insertions, recent evidence indicates that certain sequence contexts can favor insertions. Chakrabarti et al. (2019) showed that an adenine or thymine at position −4 from the PAM site increases insertion frequency [43]. This pattern is present in the effective sgRNAs targeting glutelin genes (sgGluA-1 and sgGluA-4), and in one of the prolamin-targeting sgRNAs, potentially explaining the insertion bias observed. Co-delivery of sgRNAs may further influence repair dynamics, reinforcing this effect. This confirms that CRISPR editing of multigene families can generate diverse InDel patterns depending on guide positioning and sequence context.

Protein quantification revealed significant shifts in SSP composition. In prolamin-deficient lines, the reduction in 13 kDa prolamins was coupled by increases in albumins and globulins, consistent with compensatory mechanisms previously reported [8,16]. Unlike prior RNAi studies, no significant compensation with glutelins was observed, which could be due to the retention of partial prolamin sequences (e.g., signal peptide or CCxQL motif) in some lines, potentially interfering with compensation [39]. Alternatively, the different nature of RNAi (post-transcriptional silencing) versus CRISPR (gene disruption) may lead to distinct regulatory outcomes during grain filling.

In GluA-edited lines, the response depended on the construct used. pSSLGluA-3 lines showed significant increases in prolamins and glutelins, while pSSLGluA-4 lines exhibited increased albumin and globulin content without major changes in glutelins. Interestingly, despite strong GluA suppression in pSSLGluA-3 lines (SDS-PAGE), RP-HPLC suggested elevated glutelin levels. This may reflect co-extraction of other prolamin fractions in the glutelin elution, particularly 13a, 10 kDa, or 16 kDa prolamins, as described by Shigemitsu et al. [44]. Our findings also expand on recent CRISPR-based efforts to modify rice SSP composition. For instance, Chandra et al. [21] targeted GluA-1 and GluA-2 using sgRNAs in exon 4 and observed compensatory increases in prolamins and globulins following GluA knock-out. Similarly, Pham et al. [26] edited Pro13a and Pro13b genes independently and reported reciprocal upregulation of each subgroup when the other was silenced. In our case, simultaneous disruption of both Pro13a and Pro13b abolished 13 kDa prolamins entirely, triggering a compensatory increase in albumins and globulins, but not glutelins—suggesting a saturation or threshold effect in glutelin biosynthesis. Notably, while Chen et al. [23] and Yang et al. [25] used CRISPR to target multiple glutelin genes and reported total glutelin reduction with minimal compensation, significant compensatory increases in other SSP fractions were observed in the present study; particularly in GluA-edited lines generated with pSSLGluA-3. These discrepancies may be due to differences in the target glutelin subfamilies, promoter activity of non-edited paralogs, or genotype-specific regulatory networks.

Our results suggest that compensation patterns in rice SSPs are highly dependent on which genes are edited, the structure of residual transcripts or proteins, and possibly the stage of grain development at which compensation mechanisms are activated. This highlights the value of multiplex genome editing to dissect and fine-tune these regulatory responses with subfamily-level resolution.

Remarkably, the increase in total protein content observed in most edited lines is particularly promising. To our knowledge, this is the first report of CRISPR-edited rice lines with increased total seed protein content, a trait of agronomic and nutritional relevance [3,4,45]. Although amino acid composition was not quantified in the present study, the consistent increase in lysine-rich fractions (albumins and globulins) suggests an improvement in nutritional quality. Future work should focus on amino acid profiling, detailed agronomic characterization of selected lines, transcriptomic analysis of SSP-related genes, and evaluation of processing properties in food models.

## 5. Conclusions

This study demonstrates the successful application of multiple CRISPR/Cas9 genome editing to selectively modify the composition of rice seed storage proteins. By targeting all 13 kDa prolamin and GluA glutelin subfamilies, rice lines with distinct and stable protein profiles were generated. These modifications resulted in significant changes in SSP composition, including the complete absence of specific protein fractions and the compensatory increase in others. In particular, the edited lines showed a higher accumulation of lysine-rich fractions (albumins and globulins) and, in many cases, a higher total seed protein content, highlighting their potential nutritional value. Our results also provide new insights into the different compensatory responses triggered by the disruption of specific SSP gene families and underline the importance of target selection in determining the outcome of genomic editing strategies. Overall, these findings open new approaches for engineering rice varieties with improved nutritional and functional properties through precise genome editing at the subfamily level.

## Figures and Tables

**Table 1:** CRISPR expression vectors and protospacers for rice prolamin and glutelin genes. Each vector contains two sgRNAs under different *OsU6* promoters.

Vector ID	sgRNA ID	Protospacer Sequence (5′→3′)	Length (bp)	PAM	Target
pSSLPro13-3	sgPro13a-1	GTGTTGCCAACAGCTCAGGC	20	TGG	Pro13a
	sgPro13b-1	GAAAACATCAAACTGCGCAG	20	TGG	Pro13b
pSSLGluA-3	sgGluA-1	GCAAACTGTGAAGAAAACTA	20	TGG	GluA
	sgGluA-2	GAATGGCAAAGTTCTCGCCG	20	TGG	GluA
pSSLGluA-4	sgGluA-3	GCTCGTCGTGGAAGTCCGAG	20	AGG	GluA
	sgGluA-4	GATGTCACTGATCTCAACAA	20	CGG	GluA

**Table 2:** Characterization of CRISPR/Cas9 T_0_ rice lines: Cas9 presence, number of lines analyzed for seed storage protein alterations, and number of putative edited lines.

Edited Lines	Analyzed Lines	Cas9-Positive Lines	Generation	Target	Plasmid
27	36	118	T0	Pro13b/Pro13a	pSSLPro13-3
21	27	83	T0	GluA	pSSLGluA-3
5	5	5	T0	GluA	pSSLGluA-4

**Table 3:** Characterization of CRISPR/Cas9 T_1_ rice lines: Cas9 presence, number of lines analyzed for seed storage protein alterations, and number of putative edited lines.

Edited Lines	Analyzed Lines	Lines	Generation	Target	Plasmid
9	9	27 Cas9-positives	T1	Pro13b/Pro13a	pSSLPro13-3
6	6	15 Cas9-negative			
6	6	13 Cas9-positive	T1	GluA	pSSLGluA-3
5	5	5 Cas9-negative			

## Data Availability

The original contributions presented in this study are included in the article/Supplementary Material. Further inquiries can be directed to the corresponding author.

## Supplementary Materials

The following supporting information can be downloaded at https://www.mdpi.com/article/10.3390/plants14152355/s1, Figure S1: Multiple sequence alignment of EYI105 glutelin contigs performed using ClustalOmega software version 1.2.4. The locations of sgRNAs in the consensus sequence, indicating their positions but not their sequences, are highlighted in blue; Figure S2: Multiple sequence alignment of EYI105 prolamin contigs performed using ClustalOmega software. The locations of sgRNAs in the consensus sequence, indicating their positions but not their sequences, are highlighted in blue; Figure S3: Number of sgRNAs targeting the glutelin A contigs identified in EYI105 rice cultivar with a maximum of 2 mismatches; Figure S4: Examples of SDS-PAGE total SSP profile of T2 half-seeds derived from T1 lines transformed with (a) pSSLGluA-3, and (b) pSSLPro13-3. The arrows indicate the absence of bands in edited lines. A schematic representation of the gel bands of glutelin acidic subunits is depicted in a black box with dashed lines [11]. SSP: seed storage protein. WT: wild type; Figure S5: (a) Illustrative examples of InDels in sgPro13a-1 in contigs 3 of EYI105 prolamins. (b) Illustrative examples of InDels in sgPro13b-1 in contig 5 of EYI105 prolamins; Figure S6: (a) Frequency of InDels in Sanger clones, expressed as a percentage, and calculated by dividing the total InDel events within each sgRNA by the overall number of mutations identified in 13 kDa prolamin genes of prolamin-edited lines. (b) Frequency of InDels in Sanger clones, expressed as a percentage, and calculated by dividing the total InDel events within each sgRNA by the overall number of mutations identified in glutelin genes of glutelin-A-edited lines. InDels: insertions and deletions; Figure S7: Examples of RP-HPLC prolamin profile in edited lines and WT. Retention times and mAU values are indicated for each chromatographic peak. The WT profile is represented with dashed lines to facilitate comparison. WT: wild type; Table S1: List and sequence of primers for PCR analysis and Cas9 screening of transformed lines; Table S2. List and sequences of all prolamin and glutelin Sanger amplicons from EYI105, detailing the clone’s name, corresponding line, type of InDel identified, EYI105 contig to which each amplicon aligns, and the corresponding most similar gene from the Nipponbare reference genome; Table S3: Assembled contigs of 13 kDa prolamin and GluA glutelin gene families used for sgRNA design and InDel detection in rice cultivar EYI105; Table S4: Number of glutelin amplicons sequenced and edited for each EYI105 contig in the glutelin-edited lines; Table S5: Number of prolamin amplicons sequenced and edited for each EYI105 contig in the prolamin-edited lines.

## Abbreviations

The following abbreviations are used in this manuscript:

SPPs	Seed storage proteins
RNAi	RNA of interference
sgRNAs	Single-guided ARNs
CDS	Coding Sequence
MS	Murashige and Skoog
InDels	Insertions and deletions
DTT	Dithiothreitol
RT	Room temperature
BSA	Bovine serum albumin
