# Supplementary material for: Multiplex CRISPR/Cas9 Editing of Rice Prolamin and GluA Glutelin Genes Reveals Subfamily-Specific Effects on Seed Protein Composition
Source: Plants (Basel). 2025 Jul 31;14(15):2355. doi: 10.3390/plants14152355 (PMC12349274; doi:10.3390/plants14152355)
Supplement: Supplementary file 1 [file plants-14-02355-s001.zip › Figure S1_MAS prolamins.pdf]

```
                                                                   sgPro13b-1
Consensus ATGAAGATCA TTTTCGTCTT TGCTCTCCTT GCTATTGCTG CATGCAGCGC CTCTGCGCAG   60
Contig 1  ATGAAGATCA TTTTCGTCTT TGCTCTCCTT GCTATTGCTG CATGCAGCGC CACWGCGCAG   60
Contig 5  ATGAAGATCA TTTTCGTMTT TGCTCTTCTT GCTATTGCTG CWTGCAGCGC CTCTGCGCAG   60
Contig 2  ATGAAGATCA TTTTCGTCTT TGCTCTCCTT GCTATTGCTG CATGCAGCGC CTCTGCGCAG   60
Contig 3  ATGAAGATCA TTTTCGTMTT TGCTCTCCTT GCTATTGTTG CATGCAATGC CTCTGCGCGG   60
Contig 6  ATGAAGATCA TTTTCGTMTT TGCTCTCCTT GCTATTGTTG CATGCAACGC TTCTGCACGG   60
Contig 4  ATGAAGATCA TTTTCKTMTT TGCTCTCCTT GCTATTGCTG CATGCAGCGC CTCTACGCAG   60
            sgPro13b-1
Consensus TTTGATGYTT TWRGTCAAAG TTATAGGCAA TATCAGCTGC AGTCGCATCT CCTGCTACAG  120
Contig 1  TTTGATGTTT TAGGTCAAAA TATTAGGCAA TATCAGGTGC AGTCGCCTCT CCTGCTACAG  120
Contig 5  TTTGATGTTT TAGGTCAAAG TTATAGGCAA TATCAGCTGC AGTCGCCTCT CCTGCTACAG  120
Contig 2  TTTGATGTTT TAGGTCAAAG TTATAGGCAA TATCAGCTGC AGTCGCYTST CCTGCTACAG  120
Contig 3  TTTGATCCTC TTAGTCAAAG TTATAGGCAA TATCAACTAC AGTCGCATCT CCTACTACAG  120
Contig 6  TTTGATGCTC TTAGTCAAAG TTATAGACAA TATCAACTAC AATCGCATCT CCTGCTACAG  120
Contig 4  TTTGATGCTG TTACTCATGT TTACAGGCAA TATCAGCTGC AGCCGCATCT CATGCTGCAG  120

Consensus CAACAGGTGC TTAGCCCATR YARTGAGTTC GTAAGGCAGC AGTATAGCAT AGYGGCAAGC  180
Contig 1  CAACAGGTGC TTAGCCYATA TAATGAGTTC GTAAGGCAGC AGTATAGCAT TGCGGCAAGC  180
Contig 5  CAACAGGTKC TTAGCCCATA TAATGAGTTC GTAAGGCAGG AGTATGGCAT AGCGGCAAGC  180
Contig 2  CAACAGGTGC TTAGCCCATA TAATGAGTTC GTAAGGCAGC AGTATGGCAT AGCGGCAAGC  180
Contig 3  CAACAAGTGC TCAGCCCATG CAGTGAGTTC GTAAGGCAAC AGTATAGCAT AGTGGCAACC  180
Contig 6  CAACAAGTGC TCAGCCCATG CAGTGAGTTC GTAAGGCAAC AGCATAGCAT AGTGGCAACC  180
Contig 4  CAACAGATGC TTAGCCCATG CGGTGAGTTC GTAAGGCAGC AGTGCAGCAC AGTGGAAATC  180
                                                                      sgPro13a-1
Consensus CCCTTCTTGC AATCAGCTGC GTTTCAACTG AGAAACAACC AAGTCWTGNN NNN-NNNNN-  238
Contig 1  MCCTTCTTGC AATCAGCTGY GTTTCAACTG AGAAACAACC AAGTCTTG-- ----------  228
Contig 5  CCCTTATTGC AATCAGCTGC GTTTCAACTG AGAAACAACC AWGTCTGG-- ----------  228
Contig 2  CCCTTCTTGC AATCAGCTGC GTTTCAACTG AGAAAYAACC AAGTCTGG-- ----------  228
Contig 3  CCCTTCTGGC AACCAGCTAC GTTTCAATTG ATAAACAACC AAGTCATGCA GCAGCAGTGT  240
Contig 6  CCCTTCTGGC AACCAGCTAC GTTTCAATTG ATAAACAACC AAGTCATGCA GCAACAGTGT  240
Contig 4  CCCTTCTTCC AATCACCCGT GTTTCAGCTG AGAAACTGCC AAGTCATGCA GCAGCAGTGC  240
              sgPro13a-1
Consensus NNNCAACAGC TCAGGCTGGT GGCGCAACAA TCTCACTACC AGGMCATTAR CATTGTTCAG  298
Contig 1  ---CAACAGC TCAGGCTGGT GGCGCAACAA TCTCACTACC AGGACATTAA CGTTGTCCAG  285
Contig 5  ---CAACAGC TCAGGCTGGT GGCGCAACAG TCTCACTATC AGGATATTAA AATTGTTCAG  285
Contig 2  ---CAACAGC TCGKGCTGGT GNCGCAACAA TCTCACTACC AGGACATTAA CATTGTTCAG  285
Contig 3  TGCCAACAGC TCAGGCTGGT AGCACAACAA TCTCACTACC AGGCCATTAG TATTGTTCAA  300
Contig 6  TGCCAACAGC TCAGGCTGGT AGCGCAACAA TCTCACTACC AGGCCATTAG TAGCGTTCAG  300
Contig 4  TGCCAACAGC TCAGGATGAT CGCGCAACAG TCTCACTGCC AGGCCGTTAG CAGTGTTCAG  300

Consensus GCSATWGYGC AGCAGCTACA ACTCCAGCAG TTTGGTGATC TCTACTTTGA TCRGAMTCWN  358
Contig 1  GCCATAGCGC ASCAGCTACA CCTCCAGCAG TTTGGCRATC TCTACATTGA CCGGAATCTG  345
Contig 5  GCCATAGCGC AGCAGCTGCA ACTCCAGCAG TTTGATGATC TCTACTTTGA TCGGAATCTG  345
Contig 2  GCCATAGCGC AGCAGCTACA ACTCCAGCAG TTTGGTGATC TCTACTTTGA TCGGAATCTG  345
Contig 3  GCGATTGTGC AACAGCTACA ACTGCAGCAA TTTAGTGGTG TCTACTTTGA TCAGACTCA-  359
Contig 6  GCGATTGTGC AGCAACTACA GCTGCAGCAG GTCGGTGTTG TCTACTTTGA TCAGACTCA-  359
Contig 4  GTGATTGTGC AGCAGCTACA GCTACAACAG TTTACTGGCA TCAACTTCGA TCAGACTCA-  359

Consensus NNNNNAGCTC AAGCTCAAGC TCTGTTGGCY TTWAACTTGC CATCTATATR TGGTATCTAC  418
Contig 1  GCTCAAGC-- ----TCAAGC ACTGTTGGCT TTTAACTTGC CATCTACATA TGGTATCTAC  399
Contig 5  GCTCAAGCTC AAGCTCAAGC TCTGTTGGCT TTGAACTTGC CATCTACATA TGGTATCTAC  405
Contig 2  GCTCAAGC-- ----TCAAGC TCTRTTGGCT TTTAACGTGC CATCTASATA TGGTATCTAC  399
Contig 3  -----AGCTC AAGCCCAAAC TCTGTTGACC TTCAACTTGC CATCCATATG TGGTATCTAC  414
Contig 6  -----AGCTC AAGCTCAAGC TTTGCTGGCC TTAAACTTGC CATCCATATG TGGTATCTAT  414
Contig 4  -----AGCTC TAGCCCAAGC TATGTTGGCC CTAAACTTGC CGTTAATATG CAGTATCTAC  414

Consensus CCTAGSTACT ATAGTGCWCC CAGTAGCATT ACCACCSTTG GCGGTATCTG GTACTGA     475
Contig 1  CCTWGGTMCT ATAGTGCACC BGGTAGYATT ACCACCCTTG GCGGTATCTK GTACTGA     456
Contig 5  CCTAGGTACT ATAGTGCACC TAGTAGCATT ACCACCCTTG GCGGTATCTT GTACTGA     462
Contig 2  CCTAGGTACT ATRGTGCACC CAGTACCATT ACCACCCTTG GCGGTATCTT GTACTGA     456
Contig 3  CCTAACTACT ATAGTGCTCC CAGGAGCATT GCCACTGTTG GCGGTRTCTG GTACTGA     471
Contig 6  CCTAACTACT ACATTGCTCC GAGGAGCATT CCCACCGTTG GYGGTGTCTG GTACTGA     471
Contig 4  CCAAGCTACA ACACTGCTCC CTGTAGCATT CGCACGGTCG GCGGTATCTG GTACTGA     471
```
