# Supplementary material for: Multiplex CRISPR/Cas9 Editing of Rice Prolamin and GluA Glutelin Genes Reveals Subfamily-Specific Effects on Seed Protein Composition
Source: Plants (Basel). 2025 Jul 31;14(15):2355. doi: 10.3390/plants14152355 (PMC12349274; doi:10.3390/plants14152355)
Supplement: Supplementary file 1 [file plants-14-02355-s001.zip › Figure S2_MAS glutelins.pdf]

```
Consensus GGGGTGGGAG CTTTCCCATT TGGTCATGGC ATCCATAAAT CGCCCCATAG TTATGGCATC    60
Contig 1  ---------- ---------- -----ATGGC ATCCATAAAT CGCCCCATAG TTATGGCATC    35
Contig 2  GGGGTGGGAG CTTTCCCATT TGGTCGACCT GCAGGGGGCC GCATTAGGGA TTATGGCATC    60
Contig 4  ---------- ---------- ---------- ---------- ---------- --ATGGCAAC     8
Contig 3  ---------- ---------- ---------- ---------- ---------- --ATGGCATC     8
                                   sgGluA-1
Consensus CATAAATCGC CCCATAGTTT TCTTCACAGT TTGCTTGTTC CTCTTGTGCR ATGGCTCYCT   120
Contig 1  CATAAATCGC CCCATAGTTT TCTTCACAGT TTGCTTGTTC CTCTTGTGCA ATGGCTCTCT    95
Contig 2  CATAAATCGC CCCATAGTTT TCTTCACAGT TTGCTTGTTC CTCTTGTGCG ATGGCTCCCT   120
Contig 4  CATCAAATTC CCTATAGTTT TCTCCGTCGT TTGCTTGTTC CTCTTGTGTA ATGGTTCGTT    68
Contig 3  CATAAATCGC CCCATAGTTT TCTTCACAGT TTGCTTGTTC CTCTTGTGCR ATGGCTCYCT    68
                                                  sgGluA-2    sgGluA-3
Consensus AGCCCAGCAG CTATTAGGCC AGAGCACTAG TCAATGGCAG AGTTCTCGTC GTGGAAGTCC   180
Contig 1  AGCCCAGCAG CTATTAGGCC AGAGCACTAG TCAATGGCAG AGTTCTCGTC GTGGAAGTCC   155
Contig 2  AGCCCAGCAG CTATTAGGCC AGAGCACTAG TCAATGGCAG AGTTCTCGTC GTGGAAGTCC   180
Contig 4  AGCCCAA--- CTTCTTAGCC AAAGTACTAG TCAATGGCAA AGTTCTCGCC GTGGAAGTCC   125
Contig 3  AGCCCAGCAG CTATTAGGCC AGAGCACTAG TCAATGGCAG AGTTCTCGTC GTGGAAGTCC   128
       sgGluA-3
Consensus RAGAGRATGY AGRTTYGATA GGTTGCAAGC ATTTGAGCCA ATTCGGAGTG TGAGGTCTCA   240
Contig 1  AAGAGAATGC AGGTTCGATA GGTTGCAAGC ATTTGAGCCA ATTCGGAGTG TGAGGTCTCA   215
Contig 2  GAGAGGATGT AGATTTGATA GGTTGCAAGC ATTTGAGCCA ATTCGGAGTG TGAGGTCTCA   240
Contig 4  AAGAGAGTGC AGATTTGATC GGTTGCAAGC ATTTGAGCCG ATTCGCACTG TAAGGTCCCA   185
Contig 3  RAGAGRATGY AGRTTCGATA GGTTGCAAGC ATTTGAGCCA ATTCGGAGTG TGAGGTCTCA   188

Consensus AGCTGGCACA ACTGAGTTCT TCGATGTCTC TAATGAGYWR TTTCAATGTA CCGGAGTATC   300
Contig 1  AGCTGGCACA ACTGAGTTCT TCGATGTCTC TAATGAGCAA TTTCAATGTA CCGGAGTATC   275
Contig 2  AGCTGGCACA ACTGAGTTCT TCGATGTCTC TAATGAGTTG TTTCAATGTA CCGGAGTATC   300
Contig 4  AGCTGGTACA ACTGAGTTTT TTGATGTCTC TAATGAGTTG TTTCAATGTA CTGGAGTATT   245
Contig 3  AGCTGGCACA ACTGAGTTCT TCGATGTCTC TAATGAGYWR TTTCAATGTA CCGGAGTATC   248

Consensus TGTTGTCCGY CGAGTTATTG AACCTAGAGG CCTWCTACTA CCCCATTACA CTAATGGTGC   360
Contig 1  TGTTGTCCGT CGAGTTATTG AACCTAGAGG CCTTCTACTA CCCCATTACA CTAATGGTGC   335
Contig 2  TGTTGTCCGC CGAGTTATTG AACCTAGAGG CCTACTACTA CCCCATTACA CTAATGGTGC   360
Contig 4  TGTTGTCCGT CGAGTTATCG AACCTAGAGG TCTTCTGTTA CCTCACTACT CCAATGGAGC   305
Contig 3  TGTTGTCCGY CGAGTTATTG AACCTAGAGG CCTWCTACTA CCCCATTACA CTAATGGTGC   308

Consensus ATCTCTAGTA TATATCATCC AAGGTTYGTG YAACAATTTA AGTGCATAAT GAATTAATGA   420
Contig 1  ATCTCTAGTA TATATCATCC AAGGTTCGTG CAACAATTTA AGTGCATAAT GAATTAATGA   395
Contig 2  ATCTCTAGTA TATATCATCC AAGGTTTGTG TAACAATTTA AGTGCATAAT GAATTAATGA   420
Contig 4  AACTTTGGTA TATGTCATCC AAGGTTTGTG ATTACATTTA AGTATATATT GATTAATCCT   365
Contig 3  ATCTCTAGTA TATATCATCC AAGGTTYGTG YAACAATTTA AGTGCATAAT GAATTAATGA   368

Consensus TYRGCTKCSA TRTTTAYATT GCTTGTAATT AACATGCATG CCATACTTTC AGGGAGAGGT   480
Contig 1  TCAGCTTCCA TGTTTATATT GCTTGTAATT AACATGCATG CCATACTTTC AGGGAGAGGT   455
Contig 2  TTGGCTGCGA TATTTACATT GCTTGTAATT AACATGCATG CCATACTTTC AGGGAGAGGT   480
Contig 4  TGTACTTATA T--------- CATTTTATTA ACATGGCAAT CCACACTTGC AGGCAGAGGT   416
Contig 3  TYRGCTKCSA TRTTTAYATT GCTTGTAATT AACATGCATG CCATACTTTC AGGGAGAGGT   428

Consensus ATAACAGGGC CRACTTTCCC AGGCTGTCCT GAGWCCTACC ARCARCAGTT CCAACAATCA   540
Contig 1  ATAACAGGGC CAACTTTCCC AGGCTGTCCT GAGTCCTACC AACAACAGTT CCAACAATCA   515
Contig 2  ATAACAGGGC CGACTTTCCC AGGCTGTCCT GAGACCTACC AGCAGCAGTT CCAACAATCA   540
Contig 4  ATAACAGGAC CAACTTTCCC AGGATGTCCT GAGACCTATC AACAACAGTT TCAGCAATCC   476
Contig 3  ATAACAGGGC CRACTTTCCC AGGCTGTCCT GAGWCCTACC ARCARCAGTT CCAACAATCA   488

Consensus GGSCAAGCCC AATTGACCGA AAGTCAAAGC CAAAGCCATA AGTTCAAGGA TGAACATCAA   600
Contig 1  GGCCAAGCCC AATTGACCGA AAGTCAAAGC CAAAGTCAAA AGTTCAAGGA TGAACATCAA   575
Contig 2  GGGCAAGCCC AATTGACCGA AAGTCAAAGC CAAAGCCATA AGTTCAAGGA TGAACATCAA   600
Contig 4  GAGCAAGACC AACAATTGGA AGGCCAAAGC CAAAGCCATA AATTTAGAGA TGAACATCAA   536
Contig 3  GGSCAAGCCC AATTGACCGA AAGTCAAAGC CAAAGCCATA AGTTCAAGGA TGAACATCAA   548

Consensus AAGATYCACC GTTTCAGACA AGGAGATGTW ATYGCRTTGC CTGCTGGTGT AGCTCATTGG   660
Contig 1  AAGATCCACC GTTTCAGACA AGGAGATGTA ATTGCATTGC CTGCTGGTGT AGCTCATTGG   635
Contig 2  AAGATTCACC GTTTCAGACA AGGAGATGTT ATCGCGTTGC CTGCTGGTGT AGCTCATTGG   660
Contig 4  AAGATCCACC GTTTTCAACA GGGGGATGTA GTTGCATTGC CTGCTGGTGT TGCTCATTGG   596
Contig 3  AAGATTCACC GTTTCAGACA AGGAGATGTT ATCGCGTTGC CTGCTGGTGT AGCTCATTGG   608
                                                      sgGluA-4
Consensus TGCTACAATG ATGGTGAAGT GCCRGTTGTT GCCATATATG TCACTGATAT CAACAACGGT   720
Contig 1  TGCTACAATG ATGGTGAAGT GCCAGTTGTT GCCATATATG TCACTGATCT CAACAACGGT   695
Contig 2  TGCTACAATG ATGGTGAAGT GCCGGTTGTT GCCATATATG TCACTGATAT CAACAACGGT   720
Contig 4  TGCTACAATG ATGGTGATGC ACCAATTGTT GCCATATATG TCACTGATAT ATACAATAGT   656
Contig 3  TGCTACAATG ATGGTGAAGT GCCGGTTGTT GCCATATATG TCACTGATAT CAACAACGGT   668

Consensus GCTAATCAAC TTGACCCTMG ACARAGGGTA ATTAAGCARA ACTTATCCAA AGCTAAACAT   780
Contig 1  GCTAATCAAC TTGACCCTAG GCAAAGGGTA ATTAAGCAGA ACTTTTCCAA AGCTAAACAT   755
Contig 2  GCTAATCAAC TTGACCCTCG ACAGAGGGTA ATTAAGCAAA ACTTATCCAA AACTAAACAT   780
Contig 4  GCTAACCAAC TTGATCCTAG ACACAGGGTA ATTCATCGTA TATTATCTAT AGTCATTACC   716
Contig 3  GCTAATCAAC TTGACCCTCG ACAGAGGGTA ATTAAGCAAA ACTTATCCAA AACTAAACAT   728
```

```
Consensus -TTTA----- --CTAYTATT TTGAYMTTTT TATTCCACTT TTCTTAGACA ATGATTTARC   832
Contig 1  TTTTA----- --CTACTATT TTGATATTTT TATTCCACTT ATCTTAGATG ATGATTTAGC   808
Contig 2  -TTTA----- --CTATTATT TTGACCTTTT TATTCCACTT TTCTTAGACA ATGATTTAAC   832
Contig 4  CTAAGTATTC CAATCATTTA TTTGATTTAT TGGGCAACTC TCTTTTGACA TACTAATTGT   776
Contig 3  -TTTA----- --CTATTATT TTGACCTTTT TATTCCACTT TTCTTAGACA ATGATTTAAC   780

Consensus CTYKTAATCA ATTGTTAGGA TTTCTTGTTA GCTGGAAATA AGAGAA---A CCCTCAAGCA   889
Contig 1  CTTTTAATCA ACTGTTAGGA TTTCTTGTTA GCTGGAAATA AGAGAA---A CCCTCAAGCA   865
Contig 2  CTCGTAATCA ATTGTTAGGA TTTCTTGTTA GCTGGAAATA AGAGAA---A CCCTCAAGCA   889
Contig 4  TTGTTTATGG ATTGTTAGGA TTTCTTTTTA GCTGGCAACA ATAAGATAGG TCAACAATTG   836
Contig 3  CTCGTAATCA ATTGTTAGGA TTTCTTGTTA GCTGGAAATA AGAGAA---A CCCTCAAGCA   837

Consensus TACAGGCGTG ARGTTGAGGA GYGGTCACAG AACATATTTA GTGGCTTTAG CACTGAACTR   949
Contig 1  TACAGGCGTG AGGTTGAGGA GCGGTCACAG AACATATTTA GTGGCTTTAG CACTGAACTA   925
Contig 2  TACAGGCGTG AAGTTGAGGA GTGGTCACAA AACATATTTA GTGGCTTTAG CACTGAACTG   949
Contig 4  TATAGATATG AGGCAAGGGA CAATTCGAAG AACGTCTTTG GTGGATTTAG TGTTGAACTA   896
Contig 3  TACAGGCGTG AAGTTGAGGA GYGGTCACAR AACATATTTA GTGGCTTTAG CACTGAACTR   897

Consensus CTTAGCGAGG CTCTTGGCAT AAGCARCCAA GTKGCAAGGC AGCTCCAGTG TCAAAATGAC  1009
Contig 1  CTTAGCGAGG CTCTTGGCGT AAGCAGCCAA GTGGCAAGGC AGCTCCAATG TCAAAATGAC   985
Contig 2  CTTAGCGAGG CTTTTGGCAT AAGCAACCAA GTTGCAAGGC AGCTCCAGTG TCAAAATGAC  1009
Contig 4  CTTAGCGAGG CTCTTGGCAT AAGCAGTGGA GTAGCAAGAC AACTCCAGTG CCAAAATGAC   956
Contig 3  CTTAGCGAGG CTCTTGGCGT AAGCARCCAA GTKGCAAGGC AGCTCCARTG TCAAAATGAC   957

Consensus CAAAGAGGAG AAATAGTTCG TGTTGAGCAT GGGCTTTCCT TGCTCCAACC ATATGCATCG  1069
Contig 1  CAAAGAGGAG AAAATCCCTA GTG------- ---------- ---------- ----------  1045
Contig 2  CAAAGAGGAG AAATTGTCCG CGTTGAACGC GGGCTCAGTT TGCTGCAACC ATATGCATCA  1069
Contig 4  CAAAGAGGAG AAATAGTTCG TGTTGAGCAT GGGCTTTCCT TGCTCCAACC ATATGCATCG  1016
Contig 3  CAAAGAGGAG AAATCGAATT CCCGCGGC-- ---------- ---------- ----------  1017

Consensus TTGCAAGAGC AACAACAAGA ACAGGTGCAA TCGAGAGACT ATGGCCAA-- -ACACAATAT  1126
Contig 1  ---------- ---------- ---------- ---------- ---------- ----------  1105
Contig 2  TTGCAAGAGC AGGAACAAGG ACAAATGCAA TCAAGAGAGC ATTATCAAGA AGGAGGATAT  1129
Contig 4  TTGCAAGAGC AACAACAAGA ACAGGTGCAA TCGAGAGACT ATGGCCAA-- -ACACAATAT  1073
Contig 3  ---------- ---------- ---------- ---------- ---------- ----------  1077

Consensus CAACAAAAAC AACTTCAAGG TAGTTGCTCT AATGGTTTGG ATGAGACCTT TTGTACCATG  1186
Contig 1  ---------- ---------- ---------- ---------- ---------- ----------  1165
Contig 2  CAGCAAAGTC AATATGGGAG TGGCTGCCCT AACGGTTTGG ATGAGACCTT TTGCACCATG  1189
Contig 4  CAACAAAAAC AACTTCAAGG TAGTTGCTCT AATGGTTTGG ATGAGACCTT TTGTACCATG  1133
Contig 3  ---------- ---------- ---------- ---------- ---------- ----------  1137

Consensus AGGGTAAGGC AAAATATCGA CAACCCAAAC CTCGCAGATA CATACAACCC CAGAGCAGGA  1246
Contig 1  ---------- ---------- ---------- ---------- ---------- ----------  1225
Contig 2  AGGGTAAGGC AAAACATCGA TAATCCTAAC CGTGCTGATA CATACAACCC AAGAGCTGGA  1249
Contig 4  AGGGTAAGGC AAAATATCGA CAACCCAAAC CTCGCAGATA CATACAACCC CAGAGCAGGA  1193
Contig 3  ---------- ---------- ---------- ---------- ---------- ----------  1197

Consensus AGGATCACAT ATCTAAATGG CCAAAAGTTC CCCATTCTTA ATCTTGTACA GATGAGTGCC  1306
Contig 1  ---------- ---------- ---------- ---------- ---------- ----------  1285
Contig 2  AGGGTTACAA ATCTCAACAG CCAGAATTTC CCCATTCTTA ATCTTGTACA GATGAGCGCC  1309
Contig 4  AGGATCACAT ATCTAAATGG CCAAAAGTTC CCCATTCTTA ATCTTGTACA GATGAGTGCC  1253
Contig 3  ---------- ---------- ---------- ---------- ---------- ----------  1257

Consensus GTTAAAGTAA ATTTATATCA GGTAAAACCA CATGACATTT TATCCTCATG AAAGAAAATA  1366
Contig 1  ---------- ---------- ---------- ---------- ---------- ----------  1345
Contig 2  GTTAAAGTAA ATCTATACCA GGTAAATGAA CAGCACATTT TTTTTCTTGG CAAAAGCTTT  1369
Contig 4  GTTAAAGTAA ATTTATATCA GGTAAAACCA CATGACATTT TATCCTCATG AAAGAAAATA  1313
Contig 3  ---------- ---------- ---------- ---------- ---------- ----------  1317

Consensus TTTT-GAAAA TT-----ATG CTGACAATGT TCTCTCTATT GCAGAACGCA CTCCTTTCAC  1420
Contig 1  ---------- ---------- ---------- ---------- ---------- ----------  1405
Contig 2  AATGCACACA ATATGCTTAA TGATGTGTAT CTGTTCTATT GCAGAATGCA CTCCTTTCAC  1429
Contig 4  TTTT-GAAAA TT-----ATG CTGACAATGT TCTCTCTATT GCAGAACGCA CTCCTTTCAC  1367
Contig 3  ---------- ---------- ---------- ---------- ---------- ----------  1377

Consensus CTTTTTGGAA CATCAACGCT CATAGTGTCG TGTATATTAC TCAAGG                 1466
Contig 1  ---------- ---------- ---------- ---------- ------                 1008
Contig 2  CGTTCTGGAA CATCAACGCT CACAGCATCG TGTATATTAC TCAAGG                 1475
Contig 4  CTTTTTGGAA CATCAACGCT CATAGTGTCG TGTATATTAC TCAAGG                 1413
Contig 3  ---------- ---------- ---------- ---------- ------                  985
```
