# Supplementary figures and images for: Multiplex CRISPR/Cas9 Editing of Rice Prolamin and GluA Glutelin Genes Reveals Subfamily-Specific Effects on Seed Protein Composition
Source: Plants (Basel). 2025 Jul 31;14(15):2355. doi: 10.3390/plants14152355 (PMC12349274; doi:10.3390/plants14152355)

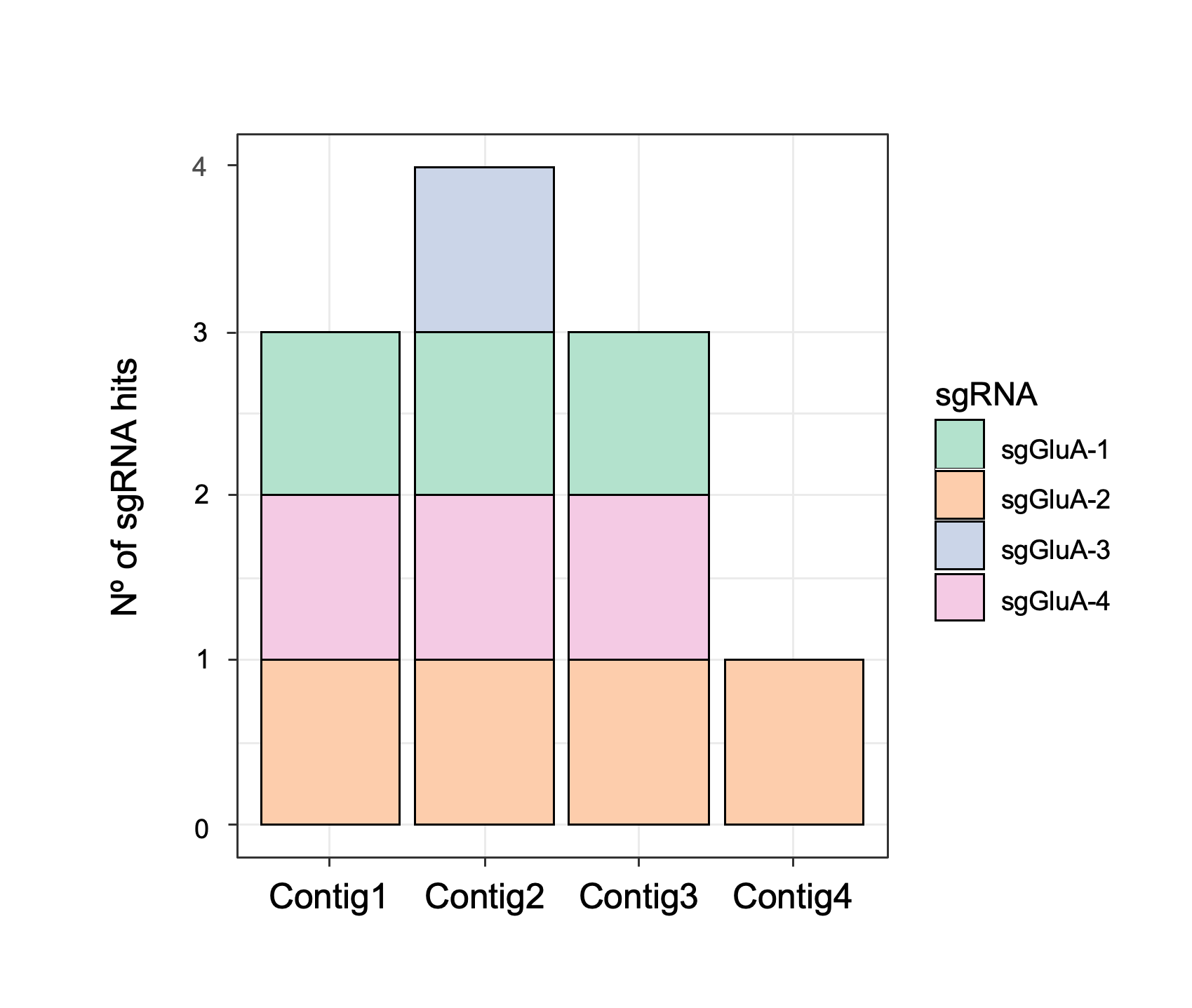

Supplement: Supplementary file 1 [file plants-14-02355-s001.zip › Figure S3_Glutelinsg RNA hits 2 mismatches.tiff]

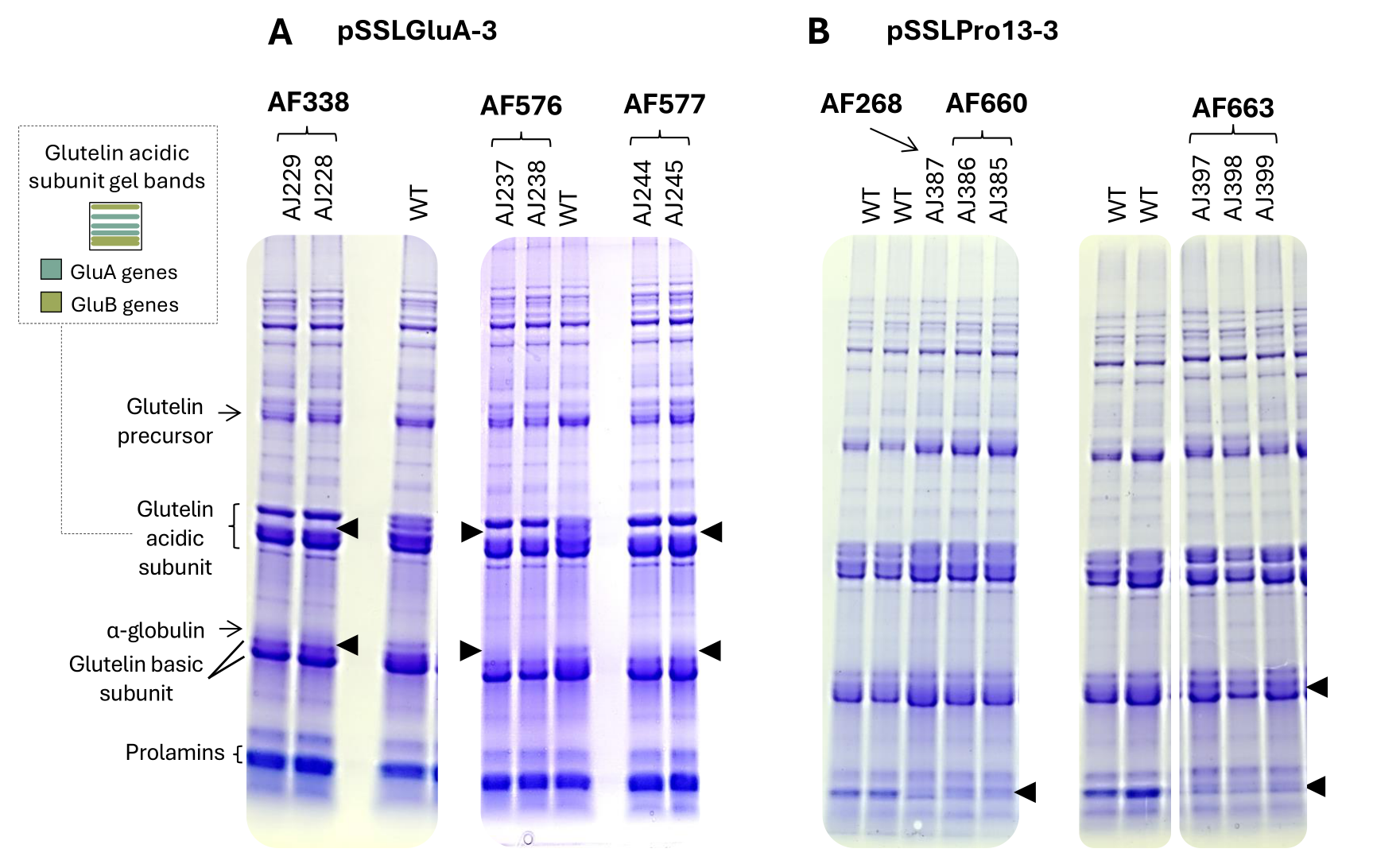

Supplement: Supplementary file 1 [file plants-14-02355-s001.zip › Figure S4_SDS-PAGET2v2.tiff]

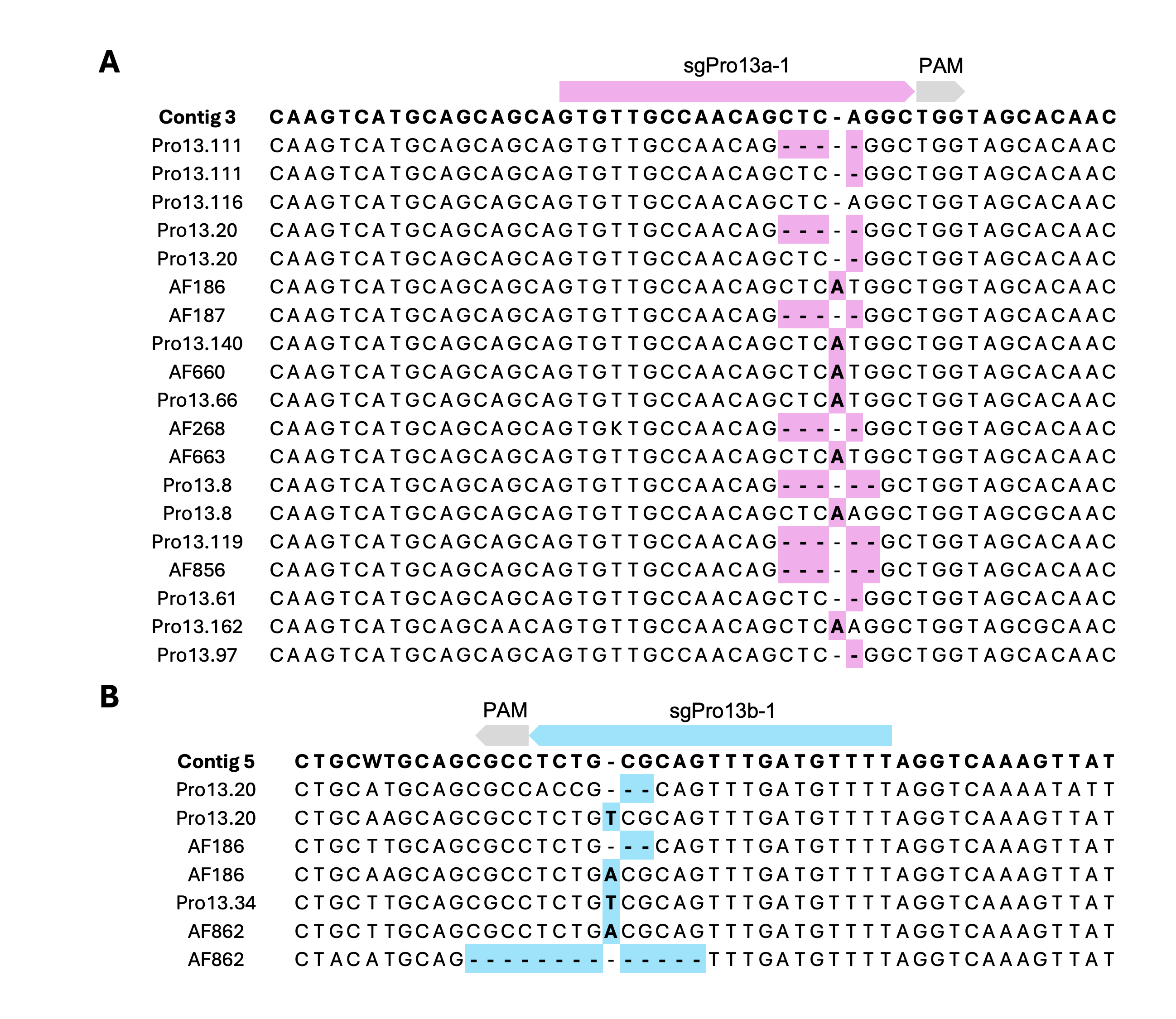

Supplement: Supplementary file 1 [file plants-14-02355-s001.zip › Figure S5_Indelsprolaminsx2.tiff]

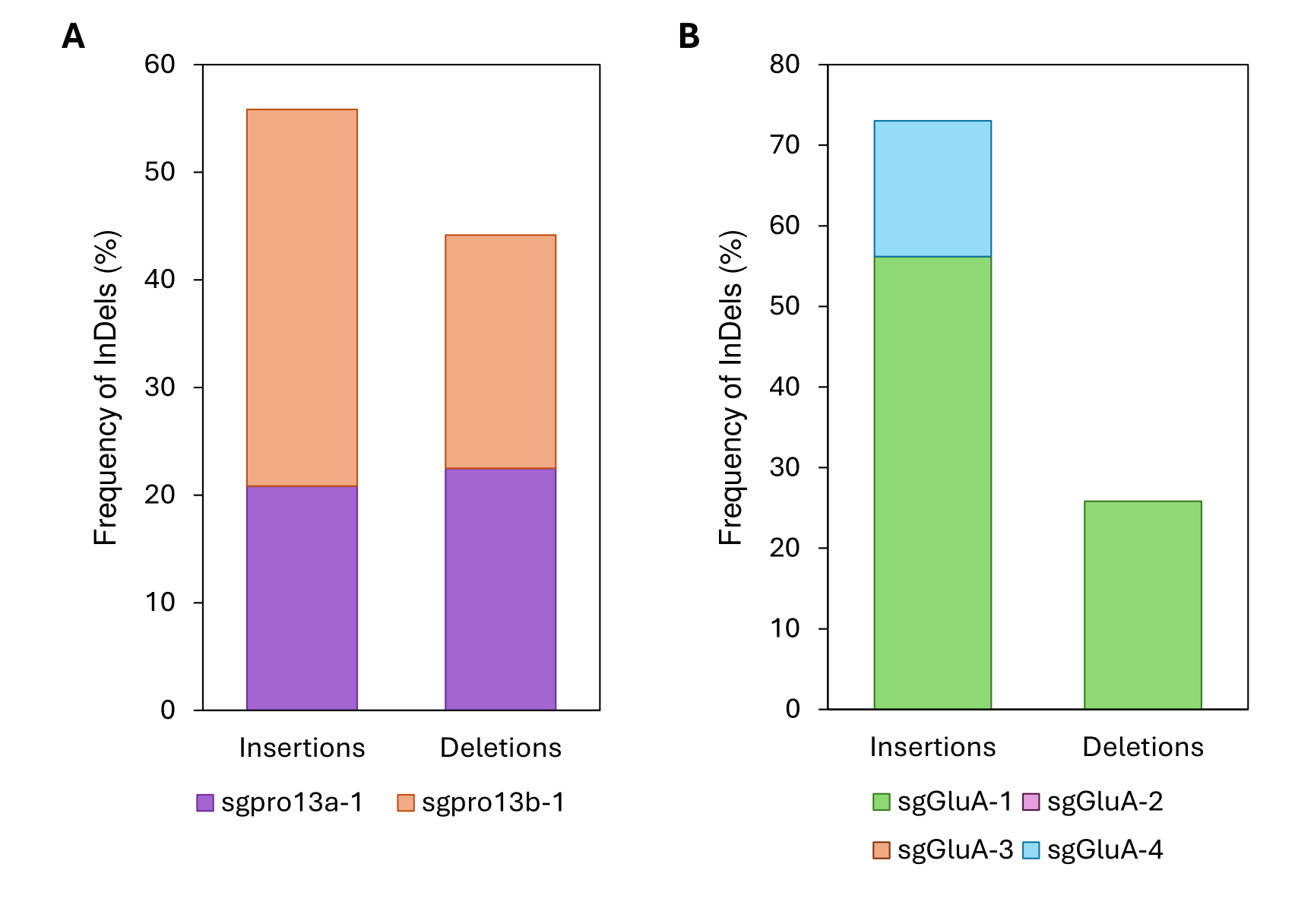

Supplement: Supplementary file 1 [file plants-14-02355-s001.zip › Figure S6_sgRNAs.tiff]

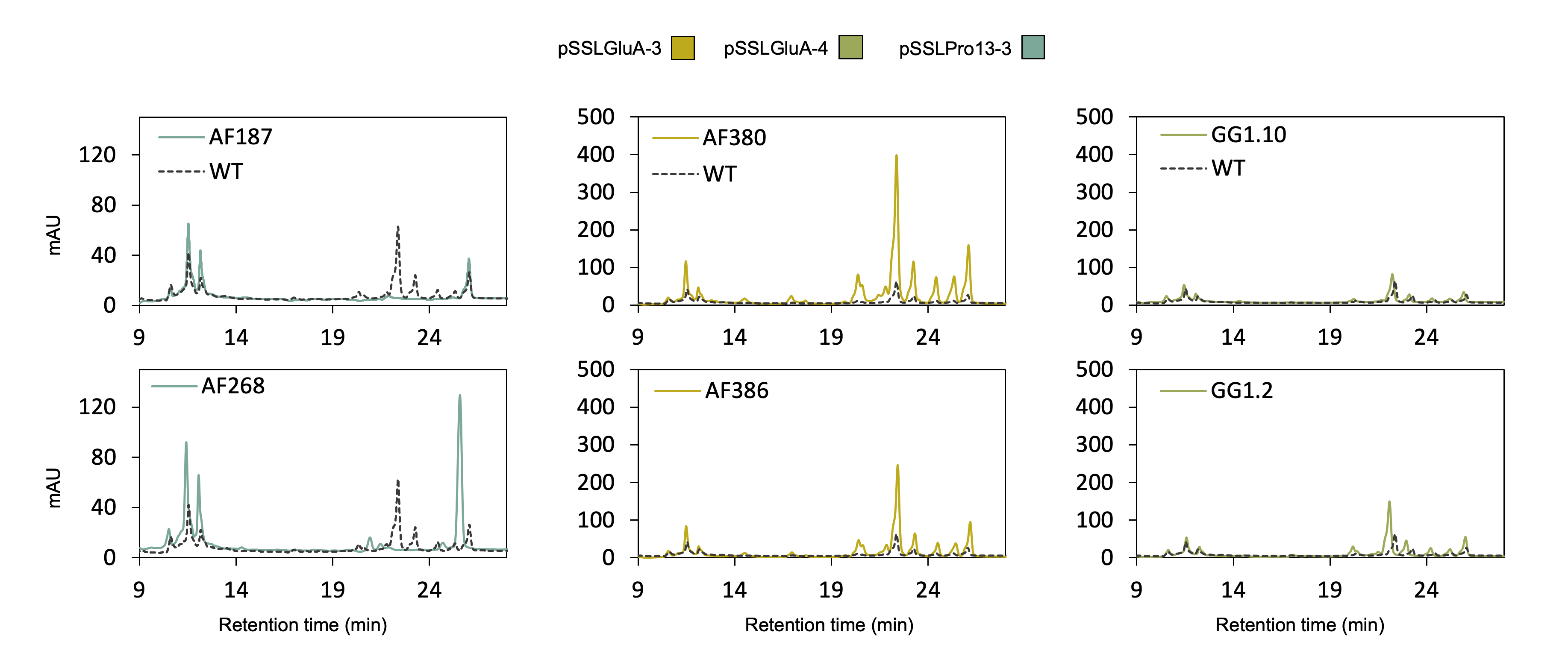

Supplement: Supplementary file 1 [file plants-14-02355-s001.zip › Figure S7_Chromatogramsv2.tiff]
